# Intelligence, &c.

**Published:** 1826-07

**Authors:** 


### INTELLIGENCE, CORRESPONDENCE, &c.

We give the following queries and speculations on a curious and obscure subject in the words of an ingenious and able practitioner of this metropolis. We leave them to the meditation of the chemical and physiological enquirers.

*Query.*—Is light, when particularly modified or combined, identical with the nervous fluid?

Whatever may be the primary source from whence life emanates, which governs, influences, and occasions the development of the animal system, yet another principle exists by which life is sustained—the whole animal machinery set in motion—and a communication formed between different parts of the system.

This effect, like magnetic and electrical action, is generally admitted to be accomplished by a fluid merely taking its name from the channels through which it flows, being termed the nervous fluid.—If our attention, in the first instance, is directed to the mode by which caloric is generated by the action of oxygen gas on carbonaceous matter, we are led to the conclusion that the air we respire produces the like effects on the blood, not entirely while it circulates within the lungs, but gradually as it passes throughout the arterial system, producing a species of subcombustion, and promoting an uniform temperature. But in combustion, (as we commonly observe it) not only is heat emitted, but a sensible quantity of light is given out. We are now led to enquire how the light is disposed of which is generated during the process of subcombustion within the blood. Light no doubt, like caloric, forms an essential constituent of oxygen gas, and, similar to it, remains in a latent state until some new combination of the oxygenous atoms set it at liberty; therefore, it is by no means improbable that, at the very period when caloric is given out, light also commences its agency—is modified—and becomes the nervous fluid. In this state it may be immediately taken up and transmitted by certain nerves giving, by ingression, the power of external sensibility—hence any mechanical impression upon them produces farther modification of this fluid, and, when transmitted to particular organs of cognizance, forms respectively the act of vision, of hearing, and of feeling, &c. &c. But although it may be said to perform these functions it is possible it fulfils others also. May not a portion be transmitted to, and condensed in, various receptacles (like electrical receivers) and be elicited in the various operations of the mind constituting, by egression, volition, and also involuntary motion? As arguments in favor of these suppositions, the galvanic action may be referred to. In the voltaic pile in proportion as the metals undergo oxydizement, so is the electric fluid given out Here again we perceive the probability that a certain portion of light remained in a latent state combined with the water used in the experiment, which, as soon as the oxygen enters into new affinities, is set free with new properties. If these ideas be entertained, we shall be induced to trace the galvanic and nervous fluids to the same origin; indeed, as the identity of these fluids is nearly established, why should not their sources be also similar.

Again, if we notice the sudden cessation or interruption of the nervous functions, if respiration be impeded or vitiated, we cannot attribute it to a defect of caloric, for this may be supplied and life still be imperfect—neither can it arise from want of action of the thorax, for this may be communicated artificially without effect. It, therefore, seems to depend on the sudden deprivation of some very active principle which cannot be retained long, only exerting its agency at the moment of creation and thus requiring constant renovation.

It may be compared to the combustion produced by immersing a platinum wire in the vapour of ether.

Light, in a latent state, may be termed lucigen, and carbonaceous combinations seem to have less capacity or affinity for it than those of hydrogen—thus, we observe the pale flame of hydrogen and the vivid light of carbon whilst undergoing combustion indicating that much latent light enters into the composition of water.

⁂ Some experiments recently made prove that violet rays of light are capable of magnetizing iron.—*R.*

---

*Expeditious Warm Bathing.* The most ingenious, and we believe the most useful, bath which we have ever seen, is that lately invented by Mr. Hicks, surgeon, of Conduit-Street, and constructed and sold by Deen, of No. 185, Regent-Street. It is of the common size, and made of copper—runs on castors—and may be readily wheeled from one room or part of a house to another. It has a kind of hollow false bottom, every where surrounded by water, into which a piece of lighted paper is thrown, and the liquid fuel turned on by means of a small cock. The flame instantly pervades the whole of this hollow bottom, and, in consequence of its intensity, and the extensive surface of application, the water in the bath is heated in ten minutes from 55 of the thermometer to upwards of 100. As the copper forming the hollow bottom is every where, excepting an aperture at each end, in contact with water, it never becomes heated beyond the temperature of the fluid, and consequently there is not the least danger of any accident to the bath. The rapidity with which a warm bath can thus be produced, is truly surprising; and is a desideratum in private houses, and, indeed, in public establishments, which has long been wanted. The expense of the fuel for heating the bath is, we understand, about one shilling. We have seen it put in operation, and recommend an examination of the bath to our medical brethren.

---

*Cupping Apparatus.* Mr. Kennedy, of Virginia Terrace, Great Dover-road, has invented a most simple and ingenious apparatus for cupping, which we cannot describe properly in words, but which may be seen by any medical gentleman at the Editor's residence at all times. This apparatus renders the operation extremely simple and easy, and, consequently, will be of great service to those practitioners in the country and colonies, who may not have all the expertness of a metropolitan cupper.

---

*Hospital Reports.* We have, on several occasions, expressed our hopes that the medical officers of public institutions in this country, metropolitan and provincial, would be induced to follow the example of their Continental brethren, in laying before the profession at large, through the medium of the press, the results of their experience in the said institutions, and thus extend the benefits of those asyla of sufferings far beyond the sphere to which they are now confined. It is with sincere satisfaction we have lately learnt that the operation of such a benevolent and useful procedure is at hand, and that, by the time this Number of our Journal has seen the light, the publication of Hospital Reports will have commenced. We are confident that no measure is more calculated to improve the practice of our profession than this—or raise it in the estimation of our brethren in other countries. One of our most respected contemporaries has announced its intention of dedicating a department to this special purpose, as will be seen by a prospectus in this Journal, and, for the present, we can only wish it success. In our next we hope to be able to add our mite of praise to the execution of the undertaking.

In the mean time, we beg to throw out one hint, the importance of which may perhaps be more apparent hereafter than at present:—it is the propriety, not to say necessity, of appointing some diligent and attentive pupil to watch and record interesting cases, under the inspection of the physician or surgeon, by which means the history will be rendered more full—the facts more authenticated—and both the pupil and the public benefited.

---

Two of our intelligent and enterprizing countrymen (Dr. Edmund Clark and Captain Sherwell) have lately conquered the formidable difficulties and dangers of the ascent to Mont Blanc, and seated themselves on the summit of that hoary king of Alps. Dr. Clark's narrative, published in the New Monthly Magazine, contains some very interesting particulars (in a medical and physiological point of view) of that arduous undertaking. We recommend the perusal of it to our professional readers.

---

*The New Edition of Laennec.* The long expected second edition of Laennec's celebrated Treatise on Diseases of the Chest, has, at length, reached this country. It is nearly double the size of the former edition, and is greatly improved in the arrangement, besides containing the entirely new department of the *treatment*. We hope to lay before our readers, in an early number, the principal novelties in these volumes; in the meantime we venture to recommend them to our readers as containing the most complete treatise on diseases of the chest, to be found in any language. We are happy to add to this notice that Dr. Forbes, the translator of the former edition, is already occupied, in preparing for the press, a second edition of his translation, containing all the improvements of the treatise above-mentioned.

---

*Reclamation.* We have received a letter from Mr. Abraham, formerly of Worcester, now of Carlisle, complaining of the unfairness of our analysis of his "Case of Sanguineous Apoplexy," published in the October number of the Edinburgh Medical Journal, and noticed at page 266 of our number (VII.) for January last. On referring to the original case and to our remarks on it, we have satisfied ourselves that we have neither garbled nor misrepresented Mr. Abraham's statements. Mr. A. has the public tribunal to appeal to, if he thinks himself wronged; and we hope he will place the original case, with our comments, in juxta-position, before that tribunal for their determination. That we have no personal feelings of unkindness to Mr. Abraham, will be evident to himself, from the manner in which we have spoken of another paper from the same pen, in the Periscope of this quarter,—which notice was sent to press long before we had received Mr. A.'s letter.

---

Our analysis of Mr. Combe's system of phrenology has been retarded by particular engagements of our correspondent who undertook the charge of concentrating the principles of that important work: his MS., though too late for insertion in the present Number, has been received, and the article shall appear in our next publication: its chief object is to shew the great practical advantages which a knowledge of the new mental philosophy is calculated to confer on the applications of medical science.

---

Mr. Henry Edmonston, of Newcastle-upon-Tyne, has in the press a letter to the Right Honourable the Earl of Liverpool concerning the present state of vaccination.

---

A medical gentleman, in genteel private practice, and most eligibly situated in the centre of the hospitals and medical schools, in the west end of the town, will take into his family one pupil, whose education he will superintend, while attending hospitals and lectures. The terms are moderate. Reference for the name and character of the gentleman may be made (by letter post paid) to Dr. James Johnson.

---

A general practitioner of considerable experience, in order to obtain a more extensive connexion than his present situation affords, wishes to purchase a share, where there might be a probability of his succeeding to the whole, of an established business.

Any gentleman having it in contemplation to retire in a few years, would find this an eligible opportunity for realizing the value of his practice. Apply, if by letter, post-paid, to Mr. Waugh, 177, Regent-street, London.

---

Mr. J. B. Baillière, of Paris, Bookseller, (Rue de l'Ecole de Médecine, No. 14,) has formed an establishment, No. 3, Bedford-street, Bedford-square, for the sale of French books of medical science, in French, German, Italian, and in other languages, which are sold at the Paris prices, No. 3, Bedford-street, Bedford-square:

| *The following are specimens of the Prices.* | £. | s. | d. |
|---|---|---|---|
| 1. Boisseau, Pyretologie, ou Traité des Fièvres, en 8vo. prix | 0 | 9 | 0 |
| 1. Bichât, Anatomie Pathologique, en 8vo. ........—— | 0 | 9 | 0 |
| 1. Leroy, Exposé pour Guerir de la Fièvre, en 8vo...—— | 0 | 4 | 0 |
| 1. Bertin, Maladie du Cœur, en 8vo ............—— | 0 | 7 | 0 |
| 1. Bouillaud, Inflammation, en 8vo. ............—— | 0 | 6 | 0 |
| 1. Bertrand, du Magnétisme en France, en 8vo. ....—— | 0 | 7 | 0 |

---

On the first of July will be published—Remarks on the late Attempt to subvert the Charter of the Royal College of Surgeons; with a dispassionate Examination of some of the Regulations of the Court. To which are subjoined, Animadversions on the evil tendency of "The Lancet," and Observations respectfully addressed to general Practitioners on the best means of maintaining their respectability and privileges. By WILLIAM COOKE, Member of the Royal College of Surgeons, Editor of an Abridgement of Morgagni's De sed. et Causis, Secretary of the Hunterian Society, &c.

---

Our readers will have seen, by the public prints, that damages have been awarded against us to the amount of one hundred pounds, for what appeared in the last number of this Journal respecting the Conductors of the Lancet. The Profession will draw their own conclusions on the event.

